# Supplementary material for: In vitro DNA SCRaMbLE
Source: Nat Commun. 2018 May 22;9:1935. doi: 10.1038/s41467-018-03743-6 (PMC5964173; doi:10.1038/s41467-018-03743-6)
Supplement: Supplementary file 1 — Supplementary Information(PDF 4549 kb) [file 41467_2018_3743_MOESM1_ESM.pdf]

In vitro DNA SCRaMbLE

Wu et al.

Supplementary Materials for

In vitro DNA SCRaMbLE

Yi Wu, Rui-Ying Zhu, Leslie A. Mitchell, Lu Ma, Rui Liu, Meng Zhao, Bin Jia, Hui Xu, Yun-Xiang Li, Zu-Ming Yang, Yuan Ma, Xia Li, Hong Liu, Duo Liu, Wen-Hai Xiao, Xiao Zhou, Bing-Zhi Li, Ying-Jin Yuan, Jef D. Boeke*

Correspondence to: Jef.Boeke@nyumc.org

This PDF file includes:

Supplementary Figure 1 to 16
Supplementary Tables 1 to 2

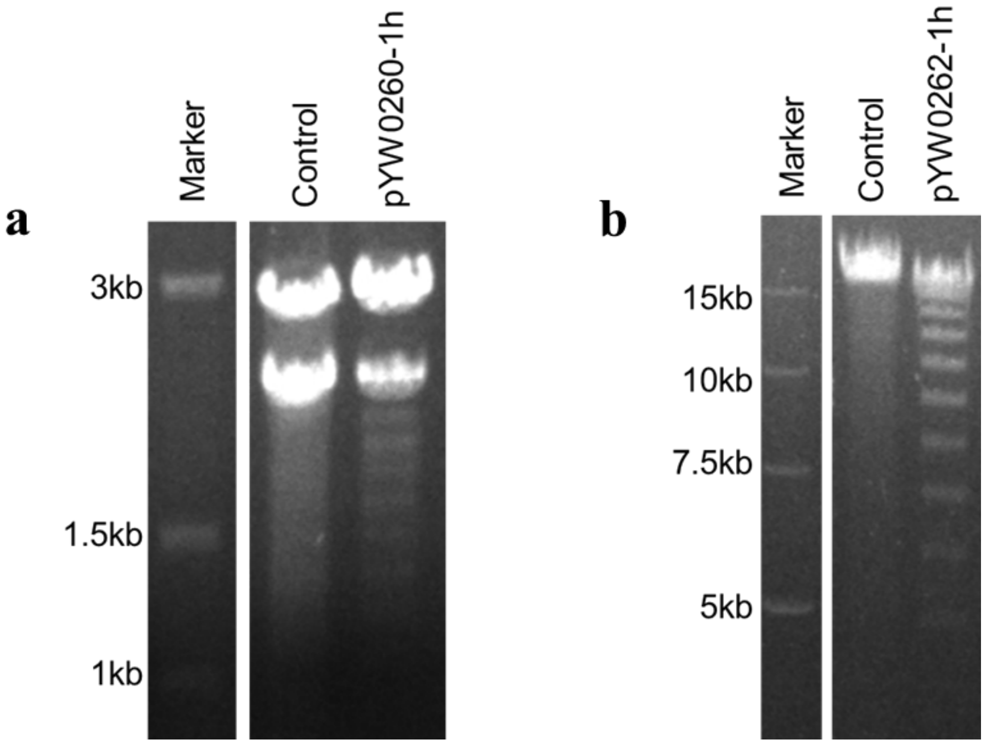

Supplementary Figure 1. ***In vitro*** **SCRaMbLEing DNA constructs encoding 10 loxPsym sites.** (a) The substrate DNA is a plasmid (pYW0260) encoding 10 loxPysm sites with 100bp between adjacent loxPsym sites. The DNA for digestion was extracted from a pool of transformed *E.coli* colonies and then linearized by *Bam*HI and *Bgl*II. (b) The substrate DNA is a plasmid (pYW0262) encoding 10 loxPysm sites with 1000bp between adjacent loxPsym sites. The DNA for digestion was extracted from population of transformed *E.coli* colonies and then linearized by *Sca*I. Marker, Trans 15k DNA Marker.

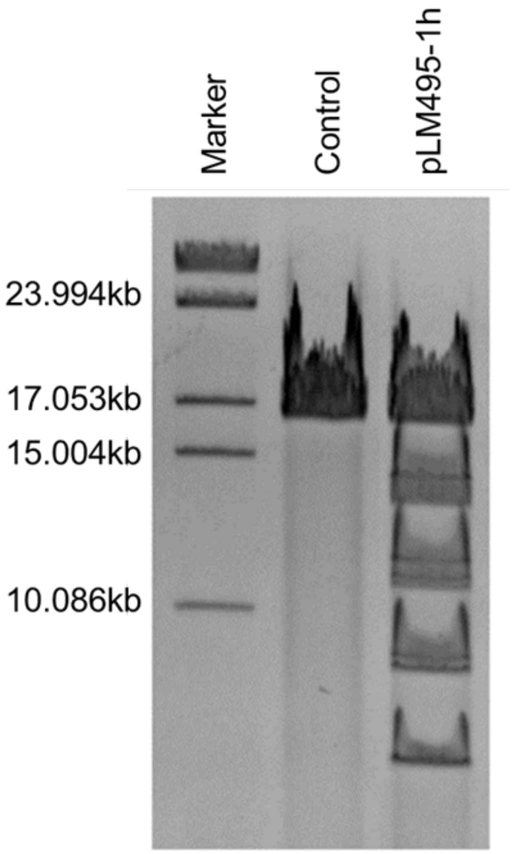

Supplementary Figure 2. Pulsed-field gel analysis of top-down *in vitro* SCRaMbLEd pLM495. The DNA libraries was extracted from pool of SCRaMblEd *E.coli* colonies and then linearized by *Not*I. Marker, Lambda DNA-Mono Cut Mix. The *Not*I site is encoded on the vector backbone of pLM495. pLM495 is 16.1kb in length with inter-loxPsym distances of 2.0, 2.6, 2.8, and 2.3 kb.

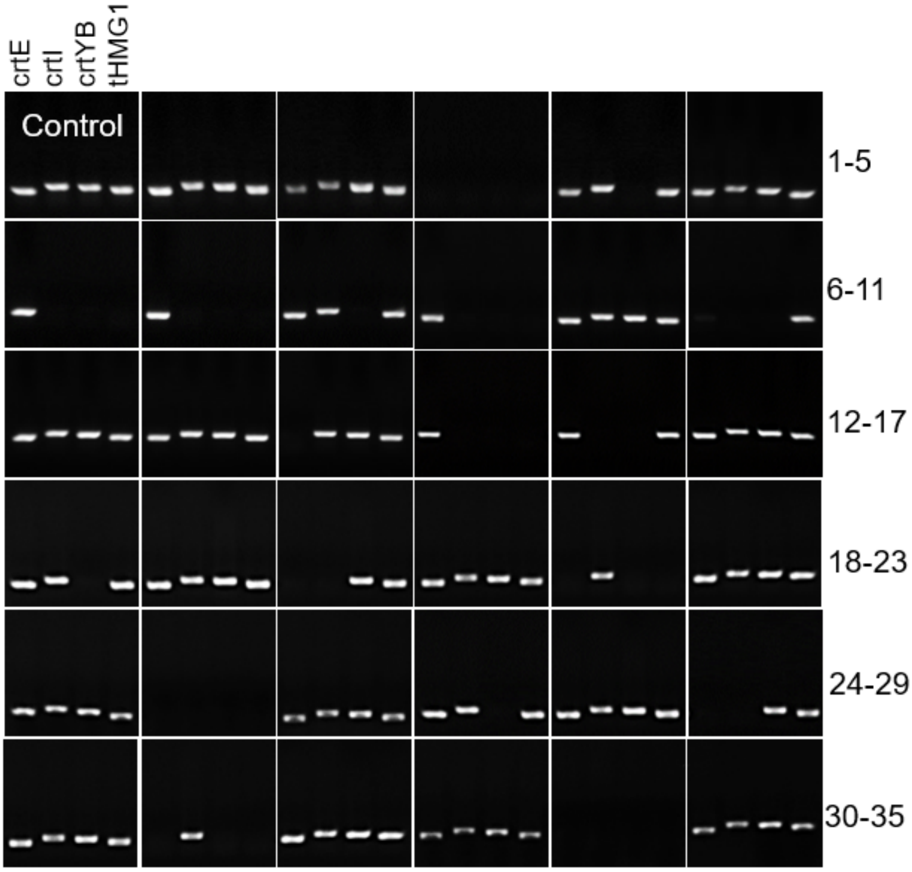

Supplementary Figure 3. PCR analysis of top-down *in vitro* SCRaMbLEd pLM495 to evaluate gene deletions. A total of 300 pLM495 *E.coli* strains (35 shown here) were randomly picked from *in vitro* SCRaMbLEd *E.coli* transformants. The control panel used purified pLM495 as template DNA.

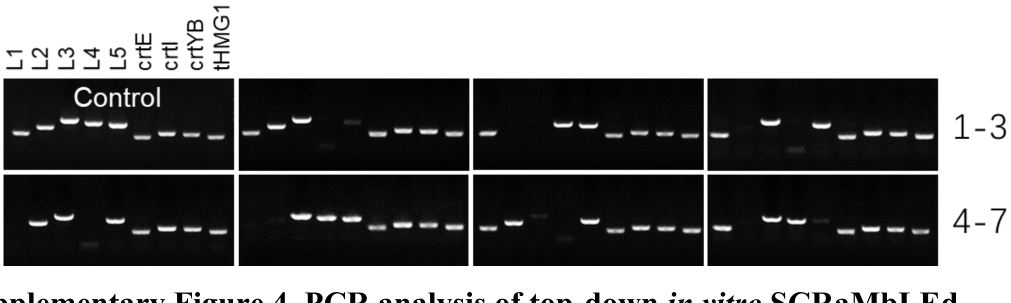

Supplementary Figure 4. PCR analysis of top-down *in vitro* SCRaMbLEd pLM495 to evaluate inversion frequency. A total of 100 pLM495 *E.coli* strains (7 shown here) were randomly picked from *E.coli* colonies transformed with *in vitro* SCRaMbLEd pLM495. L1, L2, L3, L4, L5 are specific PCR reactions designed to amplify the junction regions of vector-*crtE*, *crtE*-*crtI*, *crtI*-*crtYB*, *crtYB*-*tHMG1*, *tHMG1*-vector respectively. PCR primers for genes *crtE*, *crtI*, *crtYB*, *tHMG1* are same as supplementary figure 3.

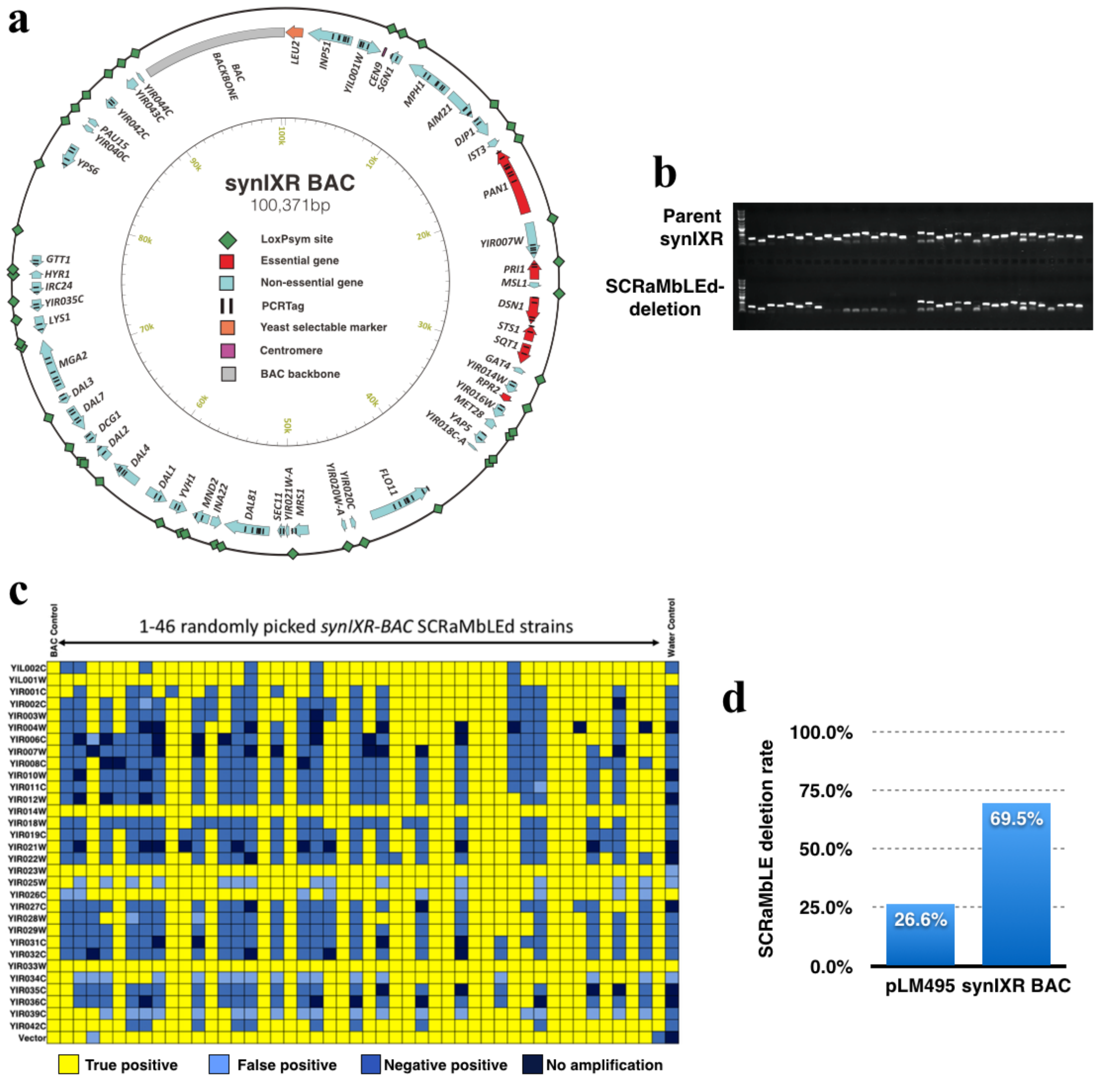

Supplementary Figure 5. Deletion frequency for top-down *in vitro* SCRaMbLEd *synIXR-BAC*. (a) Map of *synIXR-BAC*. A total of 43 loxPsym sites are encoded, along with 46 genes from chromosome *IX*. (b) PCRTag analysis to evaluate deletion events following *in vitro* SCRaMbLE. PCRTags are designed in open reading frame to distinguish synthetic sequence from wild type sequence. (c) A total of 46 *E.coli* strains were randomly picked following *in vitro* SCRaMbLE and transformation and subjected to qPCRTag analysis. A heatmap predicts amplification as a function of crossing point (Cp) in each of 1536 wells. Yellow, crossing point (Cp) value <23, defined as true positives; Light blue, 23<Cp Value<26, defined as false positive PCR products; Blue, CP value >26, defined as negative PCR products; Dark blue, no amplification. The positive values in the water control are likely primer dimers. PCRTags in the top (near YIL002C) and bottom (near YIR042C) are close to the vector sequence, which encodes the gene used to select *E. coli* transformants (beta lactamase) and are least frequently deleted. (d) Deletion frequency for *in vitro* SCRaMbLE of plasmids with 5 or 43 loxPsym sites. PCR analysis was used to count the number of deletion events after

transformation of the *in vitro* SCRaMbLEd pLM495 and *synIXR-BAC* to *E.coli* (Supplementary Figure 3, 4c).

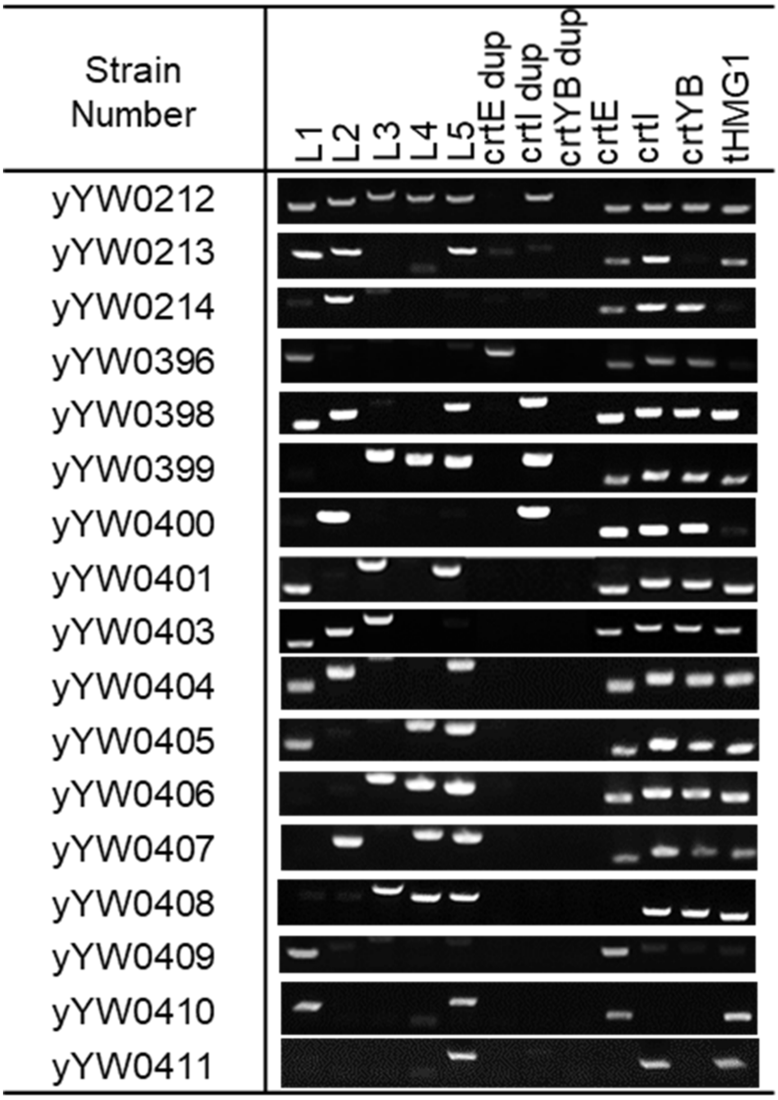

Supplementary Figure 6. PCR analysis of top-down *in vitro* SCRaMbLEd yeast strains. A total of 17 SCRaMbLEd pathways were analyzed by PCR with primers to test for deletion, inversion and duplication events. The template DNA for these reactions came from yeast recoveries as listed in Figure 2. L1, L2, L3, L4, L5 are PCR reactions designed to amplify the junction regions of vector-*crtE*, *crtE-crtI*, *crtI-crtYB*, *crtYB-tHMG1*, *tHMG1*-vector. CrtE dup, crtI dup, crtYB dup are PCR reactions designed to amplify the junction regions of duplications *crtE-crtE*, *crtI-crtI*, *crtYB-crtYB*. CrtE, crtI, crtYB, tHMG1 are PCR reactions designed to amplify individual genes *crtE*, *crtI*, *crtYB*, *tHMG1*.

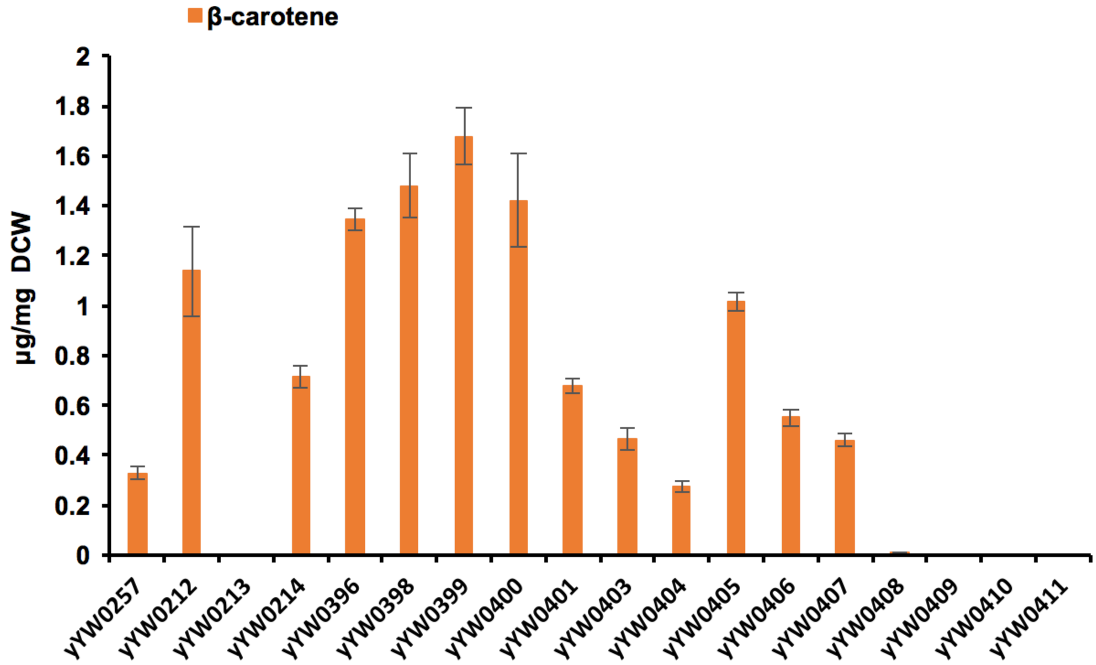

Supplementary Figure 7. HPLC measurement of β-carotene production for *in vitro* SCRaMbLEd strains. A total of 17 top-down SCRaMbLEd strains (as listed in figure 2) were tested for β-carotene production. Strain yYW0257 was a control sample with nonSCRaMbLEd pLM495. Quantification was performed in biological triplicate for each strain as shown. Error bars represent standard deviation from three replicates.

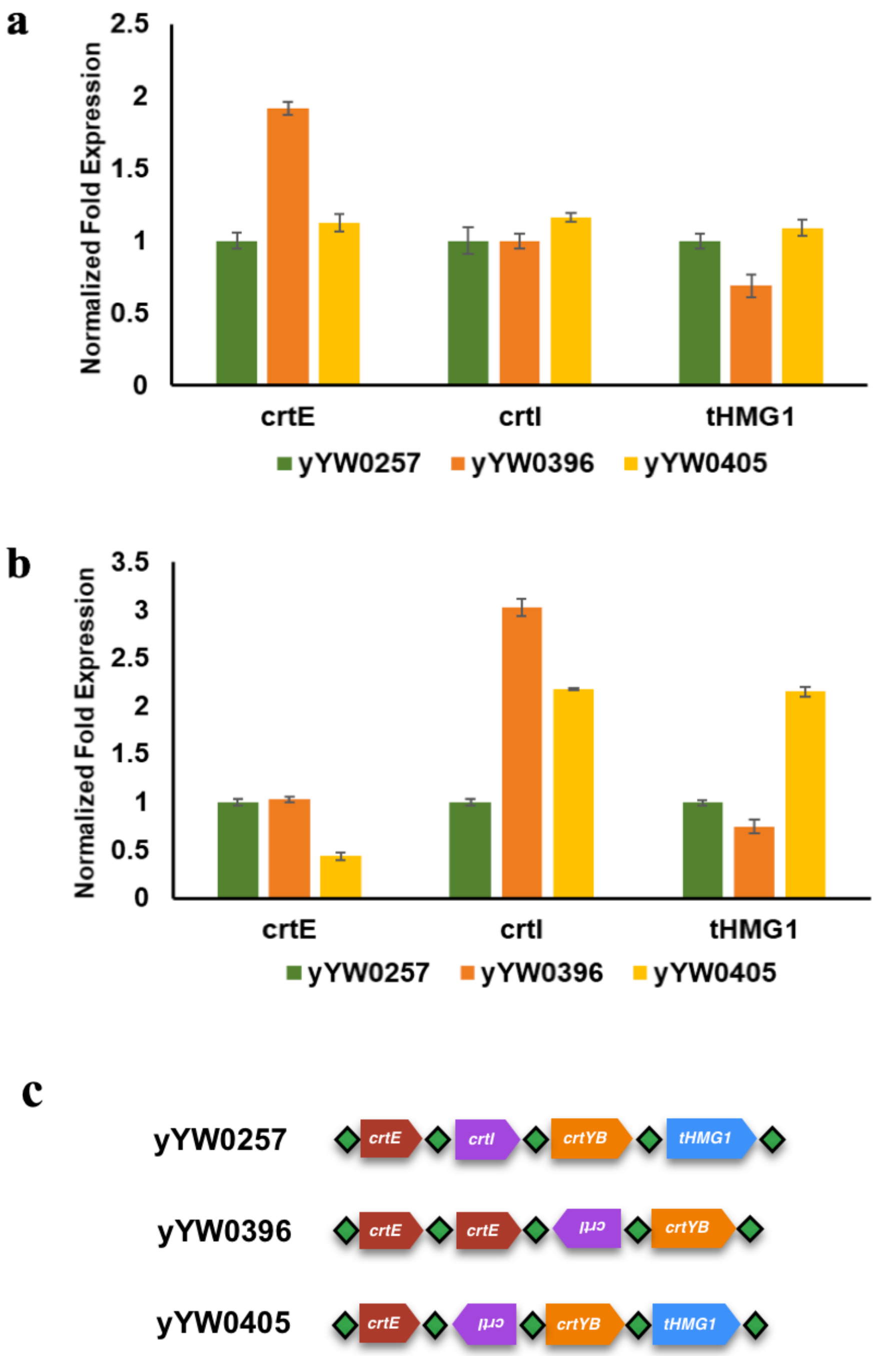

Supplementary Figure 8. qPCR analysis the inversion of *crtI* gene in the SCRaMbLEd pathway. (a) qPCR analysis of individual genes to assess copy number in the SCRaMbLEd construct. Purified yeast genomic DNA from strains carrying the SCRaMbLEd construct was used as template. (b) qPCR analysis to evaluate mRNA level for individual genes. Reverse transcribed cDNA from purified mRNA was used as templates. *CrtYB* was used as a reference gene. (c) Expected pathway structures of strains yYW0257, yYW0396, yYW0405.

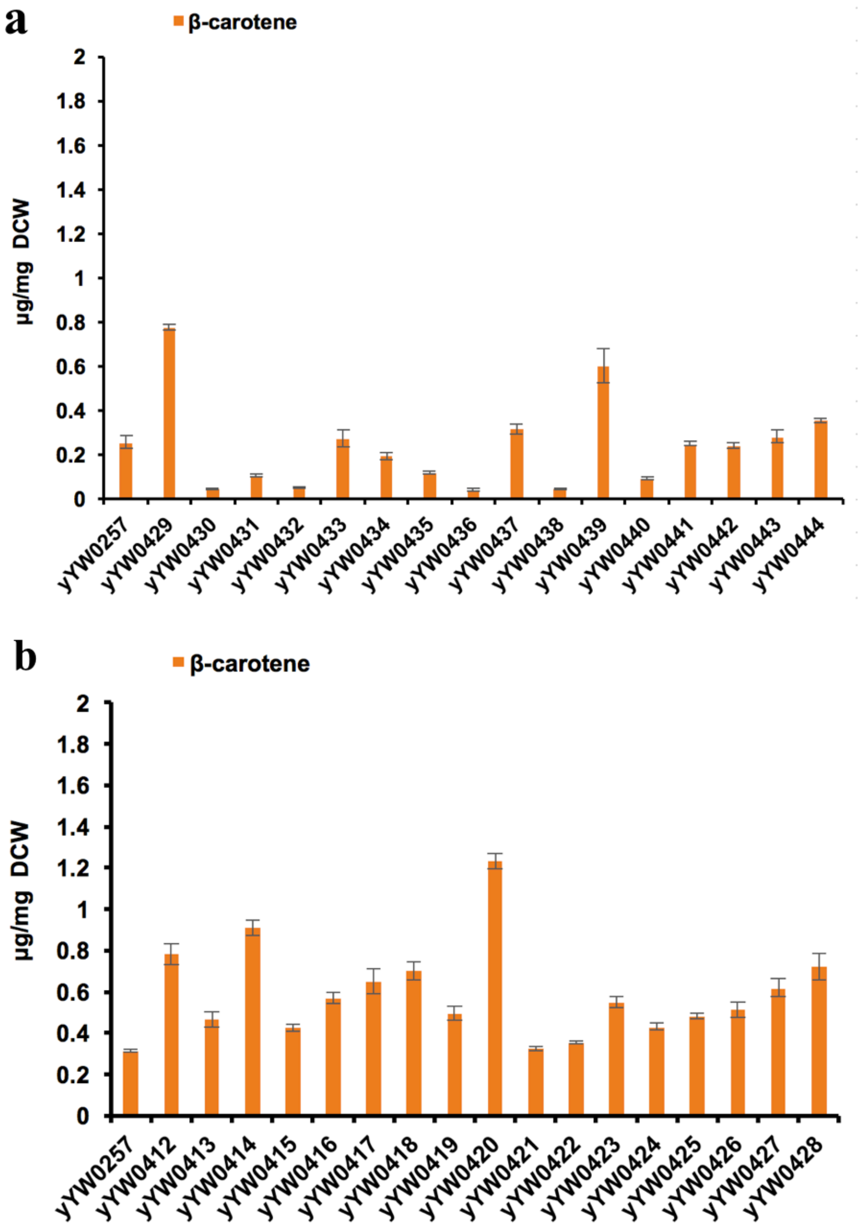

Supplementary Figure 9. HPLC measurement of β-carotene production for randomized mutation and ARTP treated strains. (a) HPLC measurement of β-carotene production of randomized mutation strains. A randomized mutation library of *crtI* gene in pLM495 was generated with a mutant rate at 5~10bp/Kb. The randomized library was transformed to *S. cerevisiae* cell for phenotypic testing. A total of 16 colonies with enhanced yellow color were screened from 1611 colonies on the plate and then subjected to measure production of β-carotene. (b) HPLC measurement of β-carotene production of atmospheric and room temperature plasma (ARTP) treated strains. A total of 17 colonies with enhanced yellow color were screened from 2353 colonies following exposure of the yeast strain yYW0257 to ARTP jet for 10 and 20 s. Strain yYW0257 was a control sample with pLM495.

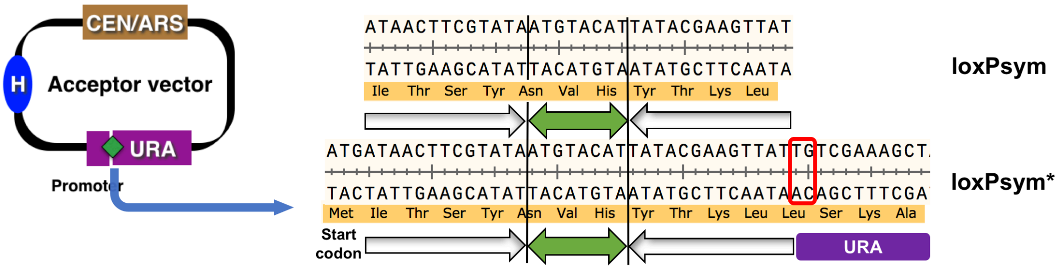

Supplementary Figure 10. Bottom-up of *in vitro* SCRaMbLE with redesigned loxPsym site. Structure and sequence comparison between loxPsym* and loxPsym. The redesigned loxPsym site (loxPsym*) is 36 bp and the insertion after start codon have no impact on the expression of *URA3*.

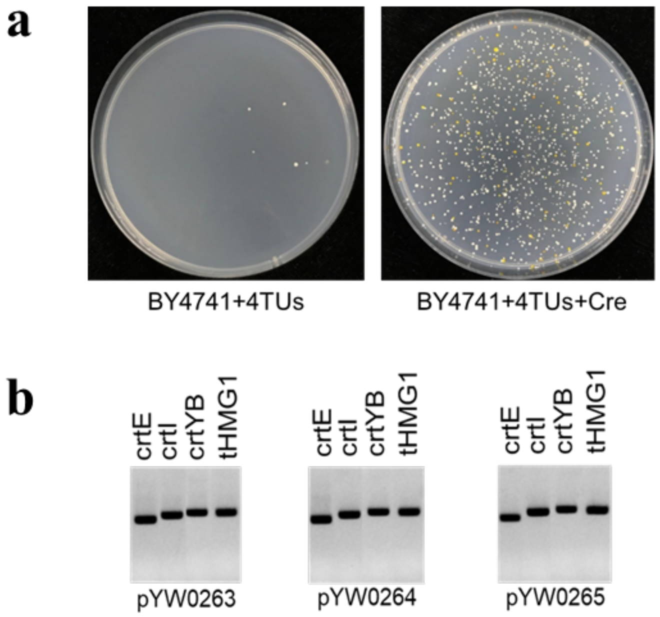

Supplementary Figure 11. Using bottom-up *in vitro* SCRaMbLE to assemble the entire β-carotene pathway from exogenous TUs. (a) BY4741, a wild-type yeast strain not carrying an integrated copy of the β-carotene pathway, was transformed with bottom-up *in vitro* SCRaMbLEd carotenogenic TU pools using strategy of the first version (left panel, Figure. 3a). Yellow colored colonies were observed on the plate. (b) PCR analysis of colored yeast colonies. The template DNA used are plasmids revovered from three colored yeast colonies. Approximately 4 percent of yeast colonies are yellow in color, suggesting bottom-up *in vitro* SCRaMbLE assembly of all four components of the β-carotene pathway.

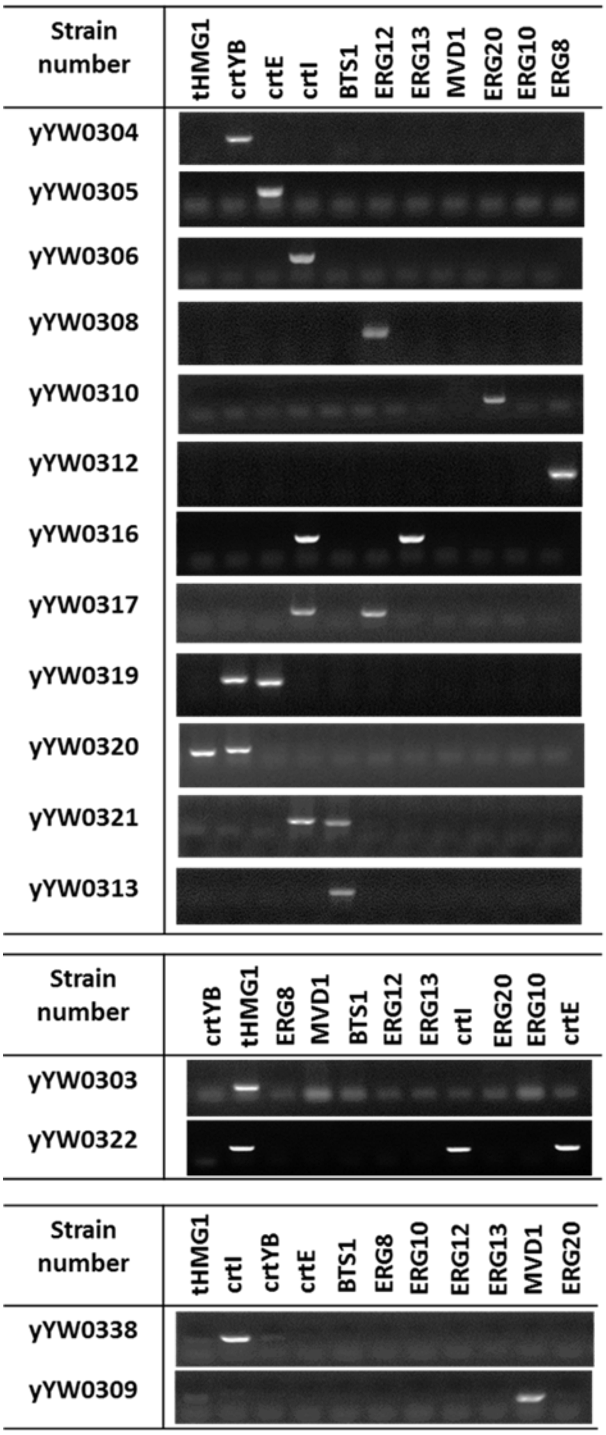

Supplementary Figure 12. PCR analysis of bottom-up SCRaMbLEd constructs in yeast. All of the bottom-up SCRaMbLEd plasmids in yeast strains listed in figure 4 were recovered to *E.coli* for PCR analysis with the primer pairs specific for the indicated genes.

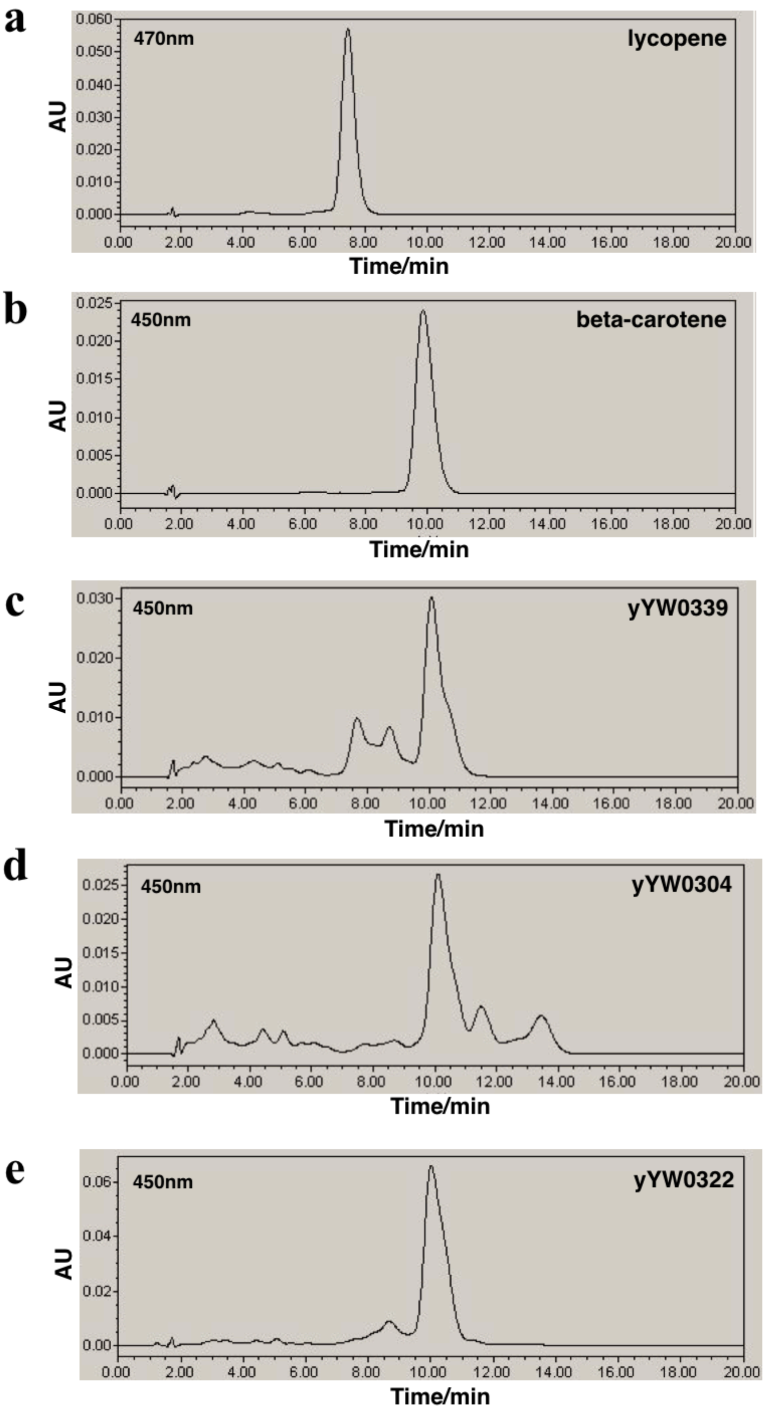

Supplementary Figure 13. HPLC profiles for bottom-up SCRaMbLEd yeast strains. (a,b) HPLC profile of standards, purified lycopene and beta-carotene; (c) HPLC curve of strain yYW0339. yYW0339 is the initial strain with non-SCRaMbLEable pathway integrated at *CAN1*; (d) HPLC curve of strain yYW0304. yYW0304 carries a SCRaMbLEd construct with an additional *crtYB* gene inserted. Two unknown peaks appear after the of β-carotene peak. (e) HPLC curve of strain yYW0322. yYW0322 carries a SCRaMbLEd construct with additional copies of three genes *crtI*, *crtE* and *tHMG1* and produces increased levels of β-carotene with minimal non-specific peaks. AU, absorbance unit.

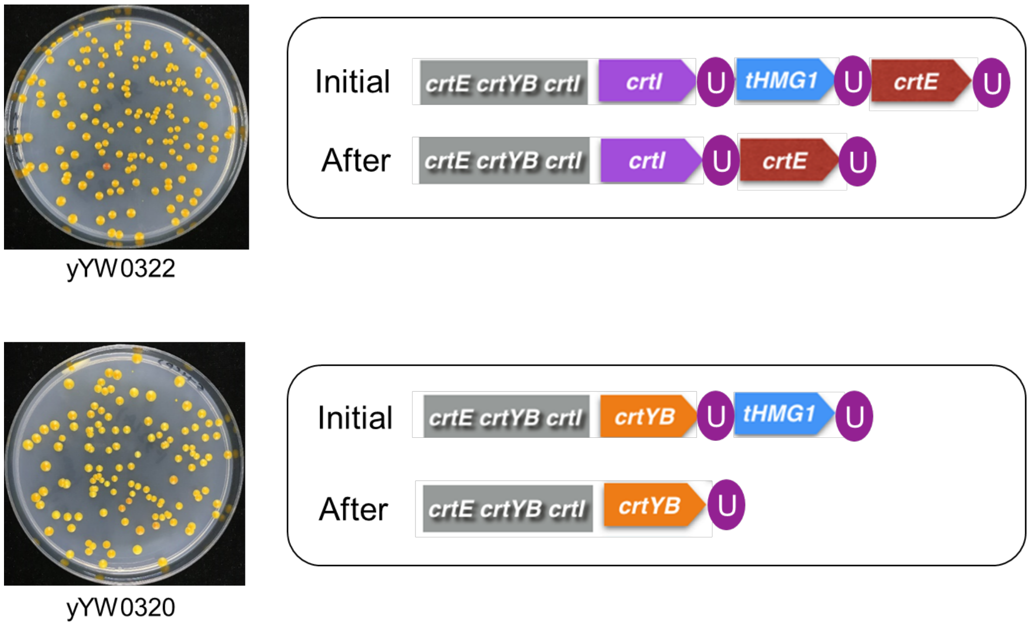

Supplementary Figure 14. Stability of bottom-up *in vitro* SCRaMbLEd constructs carrying two and three selection markers. After continuous passage for 100 generations with SCRaMbLEd strains yYW0322 and yYW0320, the recombination ratio is 1/159 for strain yYW0322 and 5/120 for strain yYW0320. The recombination events occurred between two *URA3* genes based on sequence analysis.

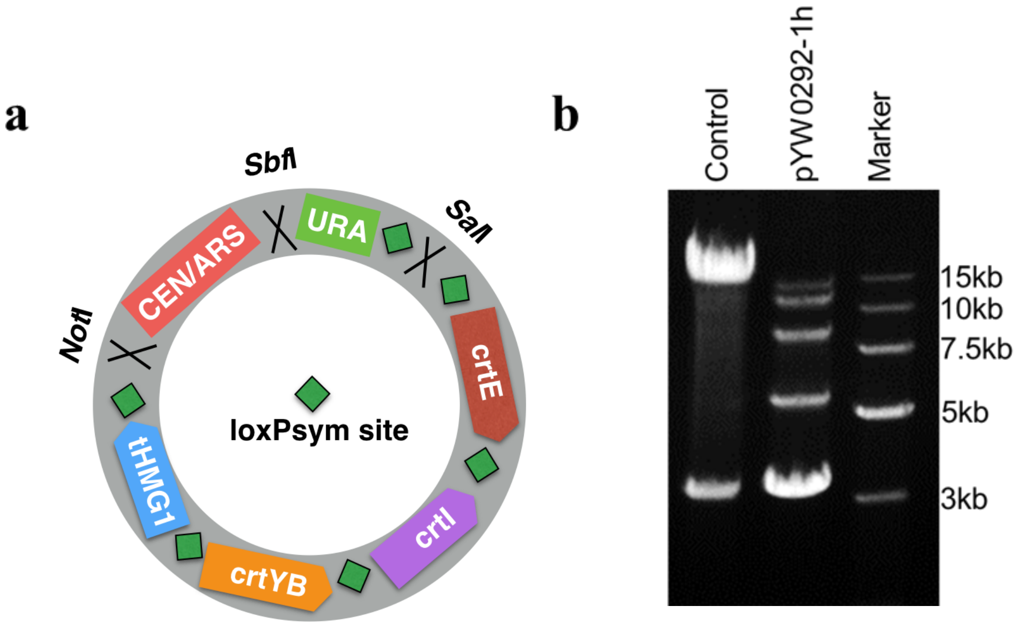

Supplementary Figure 15. Increased efficiency of *in vitro* SCRaMbLE using a redesigned pLM495 (pYW0292). (a) Map of the redesigned pLM495. A restriction enzyme cutting site (*Sal*I) was inserted between two loxPsym sites. In this way, completely unSCRaMbLEd constructs can be linearized by *Sal*I and cannot successfully transformation. (b) Agarose gel analysis of *in vitro* SCRaMbLE with pYW0292. The increased rate of truncated bands indicates increased efficiency of *in vitro* SCRaMbLE using this plasmid rather than the parental plasmid. Before transformed to *E.coli*, the SCRaMbLEd pool was digested by *Sal*I. Both control and SCRaMbLEd plasmids were digested by *Not*I and *Sbf*I before gel analysis.

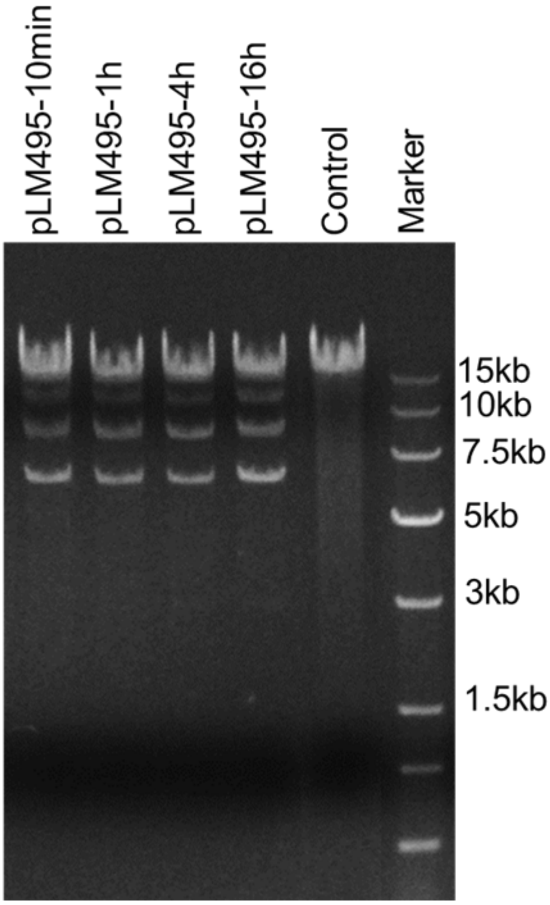

Supplementary Figure 16. Agarose gel analysis of *in vitro* SCRaMbLEd pLM495 time course. The *in vitro* SCRaMbLE reaction sampled at the indicated times. The SCRaMbLEd libraries were extracted from pool of SCRaMblEd *E.coli* colonies and then linearized by *Not*I. Marker, Trans 15k DNA Marker.

Supplementary Table 1. Yeast strains used in this study.

Strain name	Description	Genotype
BY4741		*MATa his3Δ1 leu2Δ0 met15Δ0 ura3Δ0*
yYW0257	Top-down *in vitro* SCRaMbLE control strain with unSCRaMbLEd pLM495	*MATa his3Δ1 leu2Δ0 met15Δ0 ura3Δ0*
yYW0212	Top-down *in vitro* SCRaMbLEd pLM495 yeast strain (*crtI* duplication)	*MATa his3Δ1 leu2Δ0 met15Δ0 ura3Δ0*
yYW0213	Top-down *in vitro* SCRaMbLEd pLM495 yeast strain (*crtYB* deletion)	*MATa his3Δ1 leu2Δ0 met15Δ0 ura3Δ0*
yYW0214	Top-down *in vitro* SCRaMbLEd pLM495 yeast strain (*crtI* inversion, *crtE* inversion, *tHMG1* deletion)	*MATa his3Δ1 leu2Δ0 met15Δ0 ura3Δ0*
yYW0396	Top-down *in vitro* SCRaMbLEd pLM495 yeast strain (*crtE* duplication, *crtI* inversion, *tHMG1* deletion)	*MATa his3Δ1 leu2Δ0 met15Δ0 ura3Δ0*
yYW0398	Top-down *in vitro* SCRaMbLEd pLM495 yeast strain (*crtI* duplication, *crtYB* inversion)	*MATa his3Δ1 leu2Δ0 met15Δ0 ura3Δ0*
yYW0399	Top-down *in vitro* SCRaMbLEd pLM495 yeast strain (*crtE* inversion, *crtI* duplication)	*MATa his3Δ1 leu2Δ0 met15Δ0 ura3Δ0*
yYW0400	Top-down *in vitro* SCRaMbLEd pLM495 yeast strain (*crtI* duplication, two *crtI* and *crtE* inversion, *tHMG1* deletion)	*MATa his3Δ1 leu2Δ0 met15Δ0 ura3Δ0*
yYW0401	Top-down *in vitro* SCRaMbLEd pLM495 yeast strain (*crtI* and *crtYB* inversion)	*MATa his3Δ1 leu2Δ0 met15Δ0 ura3Δ0*
yYW0403	Top-down *in vitro* SCRaMbLEd pLM495 yeast strain (*tHMG1* inversion)	*MATa his3Δ1 leu2Δ0 met15Δ0 ura3Δ0*
yYW0404	Top-down *in vitro* SCRaMbLEd pLM495yeast strain (crtYB inversion)	*MATa his3Δ1 leu2Δ0 met15Δ0 ura3Δ0*
yYW0405	Top-down *in vitro* SCRaMbLEd pLM495 yeast strain (*crtI* inversion)	*MATa his3Δ1 leu2Δ0 met15Δ0 ura3Δ0*
yYW0406	Top-down *in vitro* SCRaMbLEd pLM495 yeast strain (*crtE* inversion)	*MATa his3Δ1 leu2Δ0 met15Δ0 ura3Δ0*
yYW0407	Top-down *in vitro* SCRaMbLEd pLM495 yeast strain (*crtE* and *crtI* inversion)	*MATa his3Δ1 leu2Δ0 met15Δ0 ura3Δ0*
yYW0408	Top-down *in vitro* SCRaMbLEd pLM495 yeast strain (*crtE* deletion)	*MATa his3Δ1 leu2Δ0 met15Δ0 ura3Δ0*
yYW0409	Top-down *in vitro* SCRaMbLEd pLM495 yeast strain (*crtI crtYB* and *tHMG1* deletion)	*MATa his3Δ1 leu2Δ0 met15Δ0 ura3Δ0*
yYW0410	Top-down *in vitro* SCRaMbLEd pLM495 yeast strain (*crtI* and *crtYB* deletion)	*MATa his3Δ1 leu2Δ0 met15Δ0 ura3Δ0*
yYW0411	Top-down *in vitro* SCRaMbLEd pLM495 yeast strain (*crtE* and *crtI* deletion)	*MATa his3Δ1 leu2Δ0 met15Δ0 ura3Δ0*

yYW0301	BY4741 with β-carotene gene *crtYB, crtI, crtE* at *CAN1*	*MATa his3Δ1 leu2Δ0 met15Δ0 ura3Δ0 CAN1::crtI-crtYB-crtE-LEU*
yYW0339	Bottom-up *in vitro* SCRaMbLE control	*MATa his3Δ1 leu2Δ0 met15Δ0 ura3Δ0 CAN1::crtI-crtYB-crtE-LEU*
yYW0303	Bottom-up *in vitro* SCRaMbLEd plasmid pYW0113 with *tHMG1* inserted	*MATa his3Δ1 leu2Δ0 met15Δ0 ura3Δ0 CAN1::crtI-crtYB-crtE-LEU*
yYW0304	Bottom-up *in vitro* SCRaMbLEd plasmid pYW0113 with *crtYB* inserted	*MATa his3Δ1 leu2Δ0 met15Δ0 ura3Δ0 CAN1::crtI-crtYB-crtE-LEU*
yYW0305	Bottom-up *in vitro* SCRaMbLEd plasmid pYW0113 with *crtE* inserted	*MATa his3Δ1 leu2Δ0 met15Δ0 ura3Δ0 CAN1::crtI-crtYB-crtE-LEU*
yYW0306	Bottom-up *in vitro* SCRaMbLEd plasmid pYW0113 with *crtI* inserted	*MATa his3Δ1 leu2Δ0 met15Δ0 ura3Δ0 CAN1::crtI-crtYB-crtE-LEU*
yYW0308	Bottom-up *in vitro* SCRaMbLEd plasmid pYW0113 with *ERG12* inserted	*MATa his3Δ1 leu2Δ0 met15Δ0 ura3Δ0 CAN1::crtI-crtYB-crtE-LEU*
yYW0309	Bottom-up *in vitro* SCRaMbLEd plasmid pYW0113 with *MVD1* inserted	*MATa his3Δ1 leu2Δ0 met15Δ0 ura3Δ0 CAN1::crtI-crtYB-crtE-LEU*
yYW0310	Bottom-up *in vitro* SCRaMbLEd plasmid pYW0113 with *ERG20* inserted	*MATa his3Δ1 leu2Δ0 met15Δ0 ura3Δ0 CAN1::crtI-crtYB-crtE-LEU*
yYW0311	Bottom-up *in vitro* SCRaMbLEd plasmid pYW0113 with *ERG10* inserted	*MATa his3Δ1 leu2Δ0 met15Δ0 ura3Δ0 CAN1::crtI-crtYB-crtE-LEU*
yYW0312	Bottom-up *in vitro* SCRaMbLEd plasmid pYW0113 with *ERG8* inserted	*MATa his3Δ1 leu2Δ0 met15Δ0 ura3Δ0 CAN1::crtI-crtYB-crtE-LEU*
yYW0313	Bottom-up *in vitro* SCRaMbLEd plasmid pYW0113 with *BTS1* inserted	*MATa his3Δ1 leu2Δ0 met15Δ0 ura3Δ0 CAN1::crtI-crtYB-crtE-LEU*
yYW0316	Bottom-up *in vitro* SCRaMbLEd plasmid pYW0113 with *crtI, ERG13* inserted	*MATa his3Δ1 leu2Δ0 met15Δ0 ura3Δ0 CAN1::crtI-crtYB-crtE-LEU*
yYW0317	Bottom-up *in vitro* SCRaMbLEd plasmid pYW0113 with *crtI, ERG12* inserted	*MATa his3Δ1 leu2Δ0 met15Δ0 ura3Δ0 CAN1::crtI-crtYB-crtE-LEU*
yYW0319	Bottom-up *in vitro* SCRaMbLEd plasmid pYW0113 with *crtYB, crtE* inserted	*MATa his3Δ1 leu2Δ0 met15Δ0 ura3Δ0 CAN1::crtI-crtYB-crtE-LEU*
yYW0320	Bottom-up *in vitro* SCRaMbLEd plasmid pYW0113 with *crtYB, tHMG1* inserted	*MATa his3Δ1 leu2Δ0 met15Δ0 ura3Δ0 CAN1::crtI-crtYB-crtE-LEU*
yYW0321	Bottom-up *in vitro* SCRaMbLEd plasmid pYW0113 with *crtI, BTS1* inserted	*MATa his3Δ1 leu2Δ0 met15Δ0 ura3Δ0 CAN1::crtI-crtYB-crtE-LEU*
yYW0322	Bottom-up *in vitro* SCRaMbLEd plasmid pYW0113 with *crtE, crtI,* and *tHMG1* inserted	*MATa his3Δ1 leu2Δ0 met15Δ0 ura3Δ0 CAN1::crtI-crtYB-crtE-LEU*
yYW0338	Bottom-up *in vitro* SCRaMbLEd plasmid pYW0113 with two *crtI* inserted	*MATa his3Δ1 leu2Δ0 met15Δ0 ura3Δ0 CAN1::crtI-crtYB-crtE-LEU*
yYW0412	ARTP for yYW0257	*MATa his3Δ1 leu2Δ0 met15Δ0 ura3Δ0*
yYW0413	ARTP for yYW0257	*MATa his3Δ1 leu2Δ0 met15Δ0 ura3Δ0*
yYW0414	ARTP for yYW0257	*MATa his3Δ1 leu2Δ0 met15Δ0*

		ura3Δ0
yYW0415	ARTP for yYW0257	*MATa his3Δ1 leu2Δ0 met15Δ0 ura3Δ0*
yYW0416	ARTP for yYW0257	*MATa his3Δ1 leu2Δ0 met15Δ0 ura3Δ0*
yYW0417	ARTP for yYW0257	*MATa his3Δ1 leu2Δ0 met15Δ0 ura3Δ0*
yYW0418	ARTP for yYW0257	*MATa his3Δ1 leu2Δ0 met15Δ0 ura3Δ0*
yYW0419	ARTP for yYW0257	*MATa his3Δ1 leu2Δ0 met15Δ0 ura3Δ0*
yYW0420	ARTP for yYW0257	*MATa his3Δ1 leu2Δ0 met15Δ0 ura3Δ0*
yYW0421	ARTP for yYW0257	*MATa his3Δ1 leu2Δ0 met15Δ0 ura3Δ0*
yYW0422	ARTP for yYW0257	*MATa his3Δ1 leu2Δ0 met15Δ0 ura3Δ0*
yYW0423	ARTP for yYW0257	*MATa his3Δ1 leu2Δ0 met15Δ0 ura3Δ0*
yYW0424	ARTP for yYW0257	*MATa his3Δ1 leu2Δ0 met15Δ0 ura3Δ0*
yYW0425	ARTP for yYW0257	*MATa his3Δ1 leu2Δ0 met15Δ0 ura3Δ0*
yYW0426	ARTP for yYW0257	*MATa his3Δ1 leu2Δ0 met15Δ0 ura3Δ0*
yYW0427	ARTP for yYW0257	*MATa his3Δ1 leu2Δ0 met15Δ0 ura3Δ0*
yYW0428	ARTP for yYW0257	*MATa his3Δ1 leu2Δ0 met15Δ0 ura3Δ0*
yYW0429	Random Mutation for pLM495 of gene *crtI* (S[AGC]339S[AGU], E[GAG]368E[GAA])	*MATa his3Δ1 leu2Δ0 met15Δ0 ura3Δ0*
yYW0430	Random Mutation for pLM495 of gene *crtI* (K[AAA]9I[AUA], C[UGU]182F[UUU])	*MATa his3Δ1 leu2Δ0 met15Δ0 ura3Δ0*
yYW0431	Random Mutation for pLM495 of gene *crtI* (L[CUC]66L[CUA], D[GAU]69D[UAU])	*MATa his3Δ1 leu2Δ0 met15Δ0 ura3Δ0*
yYW0432	Random Mutation for pLM495 of gene *crtI* (P[CCU]154S[UCU], A[GCC]265V[GUC], D[GAC]267D[GAU], S[UCC]295S[UCG], Q[CAA]309H[CAU], S[AGC]339C[GGC])	*MATa his3Δ1 leu2Δ0 met15Δ0 ura3Δ0*
yYW0433	Random Mutation for pLM495 of gene *crtI* (N[AAC]41N[AAU], G[GGC]155G[GGU], I[AUC]241I[AUU], A[GCC]373V[GUC])	*MATa his3Δ1 leu2Δ0 met15Δ0 ura3Δ0*
yYW0434	Random Mutation for pLM495 of gene *crtI* (K[AAG]243D[AAC], V[GUU]322I[AUU],	*MATa his3Δ1 leu2Δ0 met15Δ0 ura3Δ0*

	G[GGU]323A[GCU], I[AUC]352I[AUU], R[CGA]384R[CGU])	
yYW0435	Random Mutation for pLM495 of gene *crtI* (L[UUG]354M[AUG])	*MATa his3Δ1 leu2Δ0 met15Δ0 ura3Δ0*
yYW0436	Random Mutation for pLM495 of gene *crtI* (I[AUC]19I[AUU], A[GCC]249A[GCU], L[CUU]261L[CUC], S[AGC]339G[GGC], V[GUG]344M[AUG], A[GCU]408S[UCU])	*MATa his3Δ1 leu2Δ0 met15Δ0 ura3Δ0*
yYW0437	Random Mutation for pLM495 of gene *crtI* (I[AUC]19V[GUC], L[UUG]354I[CUG])	*MATa his3Δ1 leu2Δ0 met15Δ0 ura3Δ0*
yYW0438	Random Mutation for pLM495 of gene *crtI* (E[GAA]122N[GAU])	*MATa his3Δ1 leu2Δ0 met15Δ0 ura3Δ0*
yYW0439	Random Mutation for pLM495 of gene *crtI* (I[AUC]19I[AUA], S[AGU]316U[ACU])	*MATa his3Δ1 leu2Δ0 met15Δ0 ura3Δ0*
yYW0440	Random Mutation for pLM495 of gene *crtI* (V[GUC]425V[GUA], A[GCA]444U[ACA])	*MATa his3Δ1 leu2Δ0 met15Δ0 ura3Δ0*
yYW0441	Random Mutation for pLM495 of gene *crtI* with no mutation	*MATa his3Δ1 leu2Δ0 met15Δ0 ura3Δ0*
yYW0442	Random Mutation for pLM495 of gene *crtI* with no mutation	*MATa his3Δ1 leu2Δ0 met15Δ0 ura3Δ0*
yYW0443	Random Mutation for pLM495 of gene *crtI* (M[AUG]82L[UUG],A[GCU]468A[GCA])	*MATa his3Δ1 leu2Δ0 met15Δ0 ura3Δ0*
yYW0444	Random Mutation for pLM495 of gene *crtI* (Y[UAU]226Y[UAC])	*MATa his3Δ1 leu2Δ0 met15Δ0 ura3Δ0*
yYW0462	Bottom-up *in vitro* SCRaMbLEd plasmid with *crtE crtI crtYB tHMG1* inserted	*MATa his3Δ1 leu2Δ0 met15Δ0 ura3Δ0 CAN1::crtI-crtYB-crtE-LEU*
yYW0463	Bottom-up *in vitro* SCRaMbLEd plasmid with *crtE crtI crtYB tHMG1* inserted	*MATa his3Δ1 leu2Δ0 met15Δ0 ura3Δ0 CAN2::crtI-crtYB-crtE-LEU*
yYW0464	Bottom-up *in vitro* SCRaMbLEd plasmid with *crtE crtI crtYB tHMG1* inserted	*MATa his3Δ1 leu2Δ0 met15Δ0 ura3Δ0 CAN3::crtI-crtYB-crtE-LEU*

Supplementary Table 2. Plasmids used in this study.

Plasmid name	Description
pLM495	Top-down initial plasmid
pYW0108	Top-down *in vitro* SCRaMbLEd pLM495, recovered from yYW0212
pYW0109	Top-down *in vitro* SCRaMbLEd pLM495, recovered from yYW0213
pYW0110	Top-down *in vitro* SCRaMbLEd pLM495, recovered from yYW0214
pYW0113	Acceptor vector
pYW0120	Universal donor vector
pYW0122	*crtI* in pYW0120
pYW0123	*crtE* in pYW0120
pYW0124	*crtYB* in pYW0120
pYW0125	*tHMG1* in pYW0120
pYW0126	*ERG10* in pYW0120
pYW0127	*ERG12* in pYW0120
pYW0128	*ERG8* in pYW0120
pYW0129	*MVD1* in pYW0120
pYW0130	*ERG20* in pYW0120
pYW0131	*BTS1* in pYW0120
pYW0198	*ERG13* in pYW0120
pYW0235	Bottom-up initial plasmid
pYW0199	Bottom-up *in vitro* SCRaMbLEd plasmid with *tHMG1* inserted, recovered from yYW0303
pYW0200	Bottom-up *in vitro* SCRaMbLEd plasmid with *crtYB* inserted, recovered from yYW0304
pYW0201	Bottom-up *in vitro* SCRaMbLEd plasmid with *crtE* inserted, recovered from yYW0305
pYW0202	Bottom-up *in vitro* SCRaMbLEd plasmid with *crtI* inserted, recovered from yYW0306
pYW0204	Bottom-up *in vitro* SCRaMbLEd plasmid with *ERG12* inserted, recovered from yYW0308
pYW0205	Bottom-up *in vitro* SCRaMbLEd plasmid with *MVD1* inserted, recovered from yYW0309
pYW0206	Bottom-up *in vitro* SCRaMbLEd plasmid with *ERG20* inserted, recovered from yYW0310
pYW0207	Bottom-up *in vitro* SCRaMbLEd plasmid with *ERG10* inserted, recovered from yYW0311
pYW0208	Bottom-up *in vitro* SCRaMbLEd plasmid with *ERG8* inserted, recovered from yYW0312
pYW0209	Bottom-up *in vitro* SCRaMbLEd plasmid with *BTS1* inserted, recovered from yYW0313
pYW0212	Bottom-up *in vitro* SCRaMbLEd plasmid with *crtI*, *ERG13* inserted, recovered from yYW0316

pYW0213	Bottom-up *in vitro* SCRaMbLEd plasmid with *crtI*, *ERG12* inserted, recovered from yYW0317
pYW0215	Bottom-up *in vitro* SCRaMbLEd plasmid with *crtYB*, *crtE* inserted, recovered from yYW0319
pYW0216	Bottom-up *in vitro* SCRaMbLEd plasmid with *crtYB*, *tHMG1* inserted, recovered from yYW0320
pYW0217	Bottom-up *in vitro* SCRaMbLEd plasmid with *crtI*, *BTS1* inserted, recovered from yYW0321
pYW0218	Bottom-up *in vitro* SCRaMbLEd plasmid with *crtE*, *crtI* and *tHMG1* inserted, recovered from yYW0322
pYW0230	Bottom-up *in vitro* SCRaMbLEd plasmid with two *crtI* inserted, recovered from yYW0338
pYW0259	36bp redesigned loxPsym* inserted after start codon of *URA3*
pYW0260	pEASY vector encoding 10 loxPysm sites with 100bp between adjacent loxPsym sites
pYW0261	pEASY vector encoding 10 loxPysm sites with 500bp between adjacent loxPsym sites
pYW0262	pEASY vector encoding 10 loxPysm sites with 1000bp between adjacent loxPsym sites
pYW0263	Recovered plasmid from yYW0263
pYW0264	Recovered plasmid from yYW0264
pYW0265	Recovered plasmid from yYW0265
pYW0268	Top-down *in vitro* SCRaMbLEd pLM495, recovered from yYW0396
pYW0270	Top-down *in vitro* SCRaMbLEd pLM495, recovered from yYW0398
pYW0271	Top-down *in vitro* SCRaMbLEd pLM495, recovered from yYW0399
pYW0272	Top-down *in vitro* SCRaMbLEd pLM495, recovered from yYW0400
pYW0273	Top-down *in vitro* SCRaMbLEd pLM495, recovered from yYW0401
pYW0275	Top-down *in vitro* SCRaMbLEd pLM495, recovered from yYW0403
pYW0276	Top-down *in vitro* SCRaMbLEd pLM495, recovered from yYW0404
pYW0277	Top-down *in vitro* SCRaMbLEd pLM495, recovered from yYW0405
pYW0278	Top-down *in vitro* SCRaMbLEd pLM495, recovered from yYW0406
pYW0279	Top-down *in vitro* SCRaMbLEd pLM495, recovered from yYW0407
pYW0280	Top-down *in vitro* SCRaMbLEd pLM495, recovered from yYW0408
pYW0281	Top-down *in vitro* SCRaMbLEd pLM495, recovered from yYW0409
pYW0282	Top-down *in vitro* SCRaMbLEd pLM495, recovered from yYW0410
pYW0283	Top-down *in vitro* SCRaMbLEd pLM495, recovered from yYW0411
pYW0292	redesigned pLM495 with *Sal*I inserted between two loxPsym sites
